# Streptomycin treatment alters the intestinal microbiome, pulmonary T cell profile and airway hyperresponsiveness in a cystic fibrosis mouse model

**DOI:** 10.1038/srep19189

**Published:** 2016-01-12

**Authors:** Mark Bazett, Marie-Eve Bergeron, Christina K. Haston

**Affiliations:** 1 Meakins Christie Laboratories and the Department of Human Genetics, McGill University, Montreal, Quebec, Canada; 2 Meakins Christie Laboratories and the Department of Medicine, McGill University, Montreal, Quebec, Canada

## Abstract

Cystic fibrosis transmembrane conductance regulator deficient mouse models develop phenotypes of relevance to clinical cystic fibrosis (CF) including airway hyperresponsiveness, small intestinal bacterial overgrowth and an altered intestinal microbiome. As dysbiosis of the intestinal microbiota has been recognized as an important contributor to many systemic diseases, herein we investigated whether altering the intestinal microbiome of BALB/c *Cftr*^tm1UNC^ mice and wild-type littermates, through treatment with the antibiotic streptomycin, affects the CF lung, intestinal and bone disease. We demonstrate that streptomycin treatment reduced the intestinal bacterial overgrowth in *Cftr*^tm1UNC^ mice and altered the intestinal microbiome similarly in *Cftr*^tm1UNC^ and wild-type mice, principally by affecting *Lactobacillus* levels. Airway hyperresponsiveness of *Cftr*^tm1UNC^ mice was ameliorated with streptomycin, and correlated with *Lactobacillus* abundance in the intestine. Additionally, streptomycin treated *Cftr*^tm1UNC^ and wild-type mice displayed an increased percentage of pulmonary and mesenteric lymph node Th17, CD8 + IL-17+ and CD8 + IFNγ+ lymphocytes, while the CF-specific increase in respiratory IL-17 producing γδ T cells was decreased in streptomycin treated *Cftr*^tm1UNC^ mice. Bone disease and intestinal phenotypes were not affected by streptomycin treatment. The airway hyperresponsiveness and lymphocyte profile of BALB/c *Cftr*^tm1UNC^ mice were affected by streptomycin treatment, revealing a potential intestinal microbiome influence on lung response in BALB/c *Cftr*^tm1UNC^ mice.

Cystic fibrosis (CF) is an autosomal recessive disease caused by mutations in the cystic fibrosis transmembrane conductance regulator (*CFTR*) gene^1^. Significant mortality and morbidity within the CF population is caused by the lung disease, which is characterized by cycles of inflammation and infection, prominently with *Pseudomonas aeruginosa* and *Staphylococcus aureus*^1^. Additionally, an estimated 40% of CF patients have airway hyperresponsiveness (AHR)^2^, the increased response of the airway to stimuli^3^,^4^. The underlying cause of the CF airway hyperresponsive phenotype is currently unknown^4^, although this trait is of relevance to CF patients, as airway response to methacholine stimulation has been correlated to decreased lung function in this population^5^,^6^ and as airway hyperresponsive CF patients were reported to have more pulmonary exacerbations and a more rapid decline in lung function^6^.

In addition to the lung disease, CF patients can develop intestinal and bone disease. Specifically, the intestinal disease manifests in CF patients as meconium ileus or distal intestinal obstructive syndrome^7^, and features small intestinal bacterial overgrowth^8^ and fecal microbial dysbiosis^9^,^10^,^11^,^12^,^13^. Bone disease, including fragile bones, reduced bone mineral density, osteoporosis^14^, and altered bone architecture^15^ can also occur in some CF patients. CF bone disease may be related to vitamin D and K deficiencies, calcium malabsoption, chronic inflammation, hormone deficiencies and CFTR dysfunction^14^, while mucus buildup, increased transit time, decreased pH and antimicrobial deficiencies have been proposed to contibute to the CF intestinal disease^16^. However, the underlying mechanisms associated with these disease manifestions are unclear^14^,^16^.

The CF traits of airway hyperresponsiveness, bone disease and intestinal disease are reflected in mice deficient for Cftr. Specifically, BALB/c *Cftr*^tm1UNC^ mice, which harbour a null mutation in *Cftr*, present with an airway hyperresponsive phenotype compared to the lung response of wild-type littermates^17^, as do FVB/N *Cftr*^tm1Eur^ mice which have the clinically prevalent delF508-Cftr mutation^18^. The airway hyperresponsive phenotype of these CF mouse models occurs in the absence of observable airway remodelling as indicated by a lack of goblet cell hyperplasia or of increased α-smooth muscle actin^17^,^18^. BALB/c *Cftr*^tm1UNC^ mice also develop a bone disease that resembles the clinical phenotype in terms of reduced bone mineral density and an altered bone structure^15^,^19^ and intestinal disease has been documented in the majority of CF mouse models^20^, including BALB/c *Cftr*^tm1UNC^ mice^21^,^22^,^23^. In CF mice the intestinal trait manifests as goblet cell hyperplasia^21^,^22^,^23^,^24^,^25^, crypt elongation^21^,^22^,^23^,^24^,^25^, increased muscle thickness^21^,^26^ and the clinically observed phenotype of small intestinal bacterial overgrowth^8^ also develops in CF mice^21^,^23^,^27^. We^21^, and others^28^, have recently reported the intestines of CF mice to harbour significant intestinal microbial dysbiosis.

The influence of the intestinal microbiome on not only intestinal^29^,^30^,^31^, but pulmonary^30^,^32^,^33^,^34^ and skeletal^35^ disease has recently been demonstrated in mouse models. In the intestine, changes to the gut microbiome have been shown to affect intestinal structure and to alter the severity of inflammatory bowel disease^29^,^30^. The potential influence of the intestinal microbiome on bone disease has been demonstrated as germ-free mice have increased bone mass compared to conventionally raised mice^35^. In the respiratory system, experimental strategies to target the intestinal microbiome, including treatment of mice with antibiotics, have shown the intestinal microbiome influence on the development of lung diseases including allergic inflammation^30^,^32^,^34^,^36^,^37^,^38^, hypersensitivity pneumonitis,^39^ and susceptibility to respiratory infections^33^,^36^,^40^. Mechanistically, these investigations point to microbiome changes which result in an altered pulmonary immune environment as leading to disease. Although the exact mechanism of how the intestinal microbiome influences the immune response is not known, effects on components of both the innate^33^,^34^,^37^ and adaptive^32^,^39^,^40^ immune system have been implicated as contributing to lung disease.

These observations have led us to hypothesize that the intestinal microbiome of Cftr deficient mice influences the development of the CF phenotypes of altered lung function, bone and intestinal disease. In the current study, we treated BALB/c *Cftr*^tm1UNC^ mice with streptomycin, a broad spectrum antibiotic not absorbed by the intestine^41^, to both decrease the bacterial load in the intestine, and to alter its microbiota, and investigated whether this intervention affected immune responses and CF traits in the lung, bones or intestine.

## Results

### Survival and body weight of Streptomycin treated mice

A population of female mice bred from heterozygous BALB/c *Cftr*^+/tm1UNC^ progenitors was maintained on streptomycin beginning *in utero*, or left untreated, until sacrifice at 12 weeks of age. At weaning at three weeks of age, 9% of the untreated mice were homozygous for *Cftr*^tm1UNC^, reflecting the reduced level of survival to weaning of CF mice, which is in agreement with previous reports of this model^21^,^23^,^42^. Of these untreated *Cftr*^tm1UNC^ mice, 26 of 34 (76%) survived to 12 weeks of age and no intestinal blockages were apparent at necropsy. Streptomycin did not significantly affect the proportion of *Cftr*^tm1UNC^ mice identified at weaning (14%, *P* = 0.07) nor their survival to 12 weeks of age (22 of 31 mice survived; 71%, *P* = 0.55). The body weight of *Cftr*^tm1UNC^ mice was significantly lower than that of wild-type mice (*P* < 0.006; Supplemental Fig. S1; weight at 12 weeks of age for *Cftr*^tm1UNC^ mice 18.5g ± 1.8; wild-type mice, 24.0g ± 3.5), in agreement with previous reports^17^,^22^,^23^,^43^, and streptomycin treatment did not affect the body weight of either group (*P* > 0.10; Supplemental Fig. S1).

### Streptomycin treatment reduced intestinal bacterial load and altered the intestinal microbiome of BALB/c *Cftr* ^*tm1UNC*^ mice

**Figure 1:**
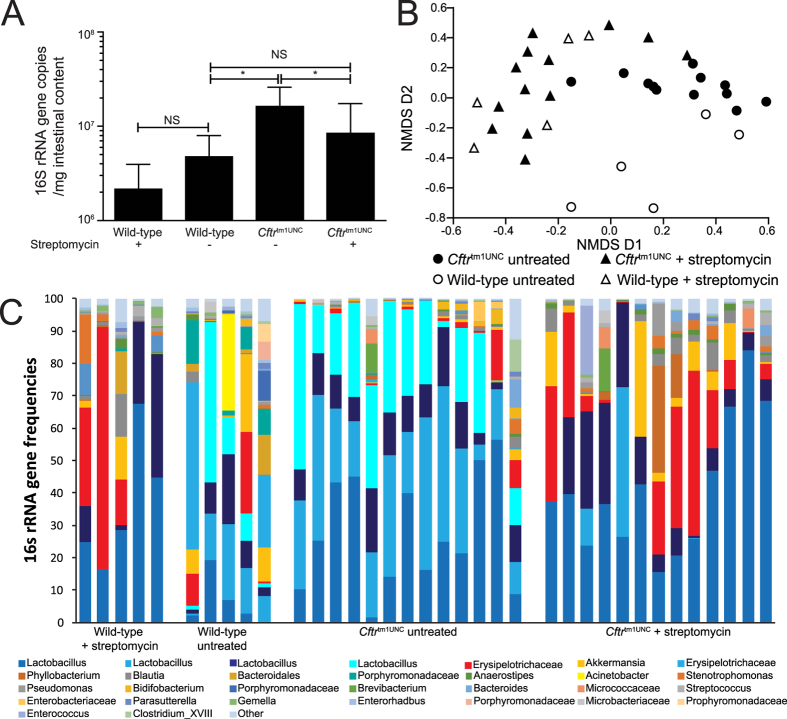
Small intestinal bacterial load and microbiome of female BALB/c *Cftr*^tm1UNC^ mice and wild-type littermates, untreated and treated with streptomycin beginning *in utero* until death at 12 weeks of age. (**A**) Bacterial load was measured using quantitative real-time PCR of the 16S rRNA of DNA isolated from 150mg of small intestinal contents. Average ± standard deviation is shown (n = 8–13 mice per group). *indicates a significant difference between groups, *P* < 0.05, by Student’s *t-*test. NS = non-significant. (**B**) Two dimensional non-metric multidimensional scaling (NMDS) of the Bray-Curtis dissimilarity between microbiome samples. (**C**) 16S rRNA gene frequencies of the most abundant operational taxonomic units (OTU) classified to the closest related taxon.

To quantify the effect of streptomycin treatment on the intestinal bacterial load and its composition, the small intestinal contents of 12 week old mice were collected and assayed using real-time PCR and pyrosequencing of the 16S rRNA gene. As shown in Fig. 1A, untreated *Cftr*^tm1UNC^ mice had significant intestinal bacterial overgrowth compared to levels in wild-type mice (*P* < 0.002), as has been documented previously in this strain^21^,^23^. Streptomycin treatment reduced the bacterial overgrowth in *Cftr*^tm1UNC^ mice (*P* = 0.01) to wild-type levels (*P* = 0.26), but did not significantly affect levels in the wild-type mice (*P* = 0.15).

Pyrosequencing confirmed the existence of microbial dysbiosis in the intestinal contents of untreated *Cftr*^tm1UNC^ mice relative to untreated wild-type mice, which has been previously documented^21^, as these groups of mice clustered separately based on analysis of community composition (Fig. 1B). In contrast, streptomycin treatment shifted the community composition of both the *Cftr*^tm1UNC^ mice and wild-type mice, as samples from these groups of mice receiving streptomycin clustered together (Fig. 1B). An evaluation of sequence diversity revealed samples from untreated wild-type mice to be more diverse than those from *Cftr*^tm1UNC^ mice (Shannon’s Diversity; *Cftr*^tm1UNC^ mice, 1.4 ± 2.2; wild-type mice, 1.96 ± 0.38; *P* = 0.026), and for streptomycin treatment not to significantly alter diversity in either of wild-type (streptomycin treated wild-type mice, 1.47 ± 0.57, *P* = 0.15 vs. untreated wild-type mice) or *Cftr*^tm1UNC^ mice (streptomycin treated *Cftr*^tm1UNC^ mice, 1.40 ± 0.38, *P* = 0.99 vs. untreated *Cftr*^tm1UNC^ mice).

To determine which intestinal bacteria were altered in abundance between mice grouped by *Cftr* genotype and treatment, we initially used phylum level classification of bacterial DNA sequences. This analysis revealed a significant difference between samples from untreated *Cftr*^tm1UNC^ and untreated wild-type mice in the abundance of bacteria from two phyla; bacteria from the phylum Firmicutes were more abundant in *Cftr*^tm1UNC^ mice (*P* = 0.009) and those from Verrucomicrobia were more abundant in wild-type mice (*P* = 0.03), as shown in Supplemental Fig. S2. Streptomycin treatment of *Cftr*^tm1UNC^ mice significantly affected the abundance of both Firmicutes (*P* = 0.03) and Verrucomicrobia (*P* = 0.002) in the intestine, compared to levels in untreated *Cftr*^tm1UNC^ mice resulting in no significant differences in bacterial abundance at the phylum level among untreated wild-type, streptomycin treated wild-type or streptomycin treated *Cftr*^tm1UNC^ mice (Supplemental Fig. S2).

OTU classification revealed differences in the abundance of particular bacteria, between untreated *Cftr*^tm1UNC^ mice and untreated wild-type mice, and these included groups of *Lactobacillus, Akkermansia,* Porphyromonadaceae, *Enterohadbus* and Coriobacteriaceae (as illustrated in Fig. 1C). Of the 50 OTUs most frequently detected in the intestinal samples, those differing significantly in abundance between untreated *Cftr*^tm1UNC^ mice and untreated wild-type mice, are listed in Table 1. Streptomycin treatment of *Cftr*^tm1UNC^ mice affected the abundance of multiple OTUs, compared to levels detected in untreated *Cftr*^tm1UNC^ mice, including those corresponding to *Lactobacillus* and *Akkermansia,* among others presented in Table 2. With the exception of OTU49 (*Staphylococcus*), there were no significant differences in bacterial abundance between streptomycin treated *Cftr*^tm1UNC^ mice and streptomycin treated wild-type mice for the 50 most abundant OTUs, supporting the convergence in microbiome composition after streptomycin treatment depicted in Fig. 1.

### Streptomycin treatment did not affect the intestinal or bone disease of BALB/c *Cftr* ^*tm1UNC*^ mice

**Figure 2:**
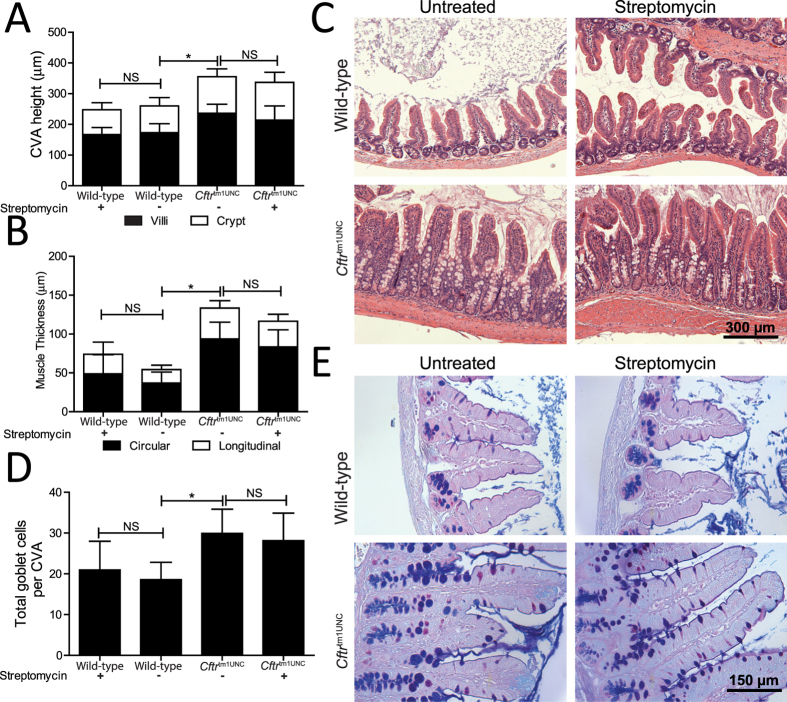
Architecture of the small intestine in female BALB/c *Cftr*^tm1UNC^ mice and wild-type littermates, untreated and treated with streptomycin beginning *in utero* until death at 12 weeks of age. (**A**) Crypt to Villus axis height (CVA) was measured by image analysis of histological sections for 25 ileal CVAs per mouse. (**B**) Muscularis externa thickness was measured by image analysis of histological sections. (**C**) Representative ileal sections showing CF CVA distention and muscle thickness increase in BALB/c *Cftr*^tm1UNC^ mice. Hematoxylin and Eosin stain, magnification 200X. (**D**) Total goblet cells per CVA were measured by image analysis of histological sections for 25 ileal CVAs per mouse. (**E**) Representative ileal sections showing CF goblet cell number increase. Periodic acid-Schiff/Alcian Blue stain, magnification 400X. Data presented as the mean ± standard deviation (n = 7–9 mice per group). *indicates a significant difference between groups, *P* < 0.05, by Student’s *t-*test. NS = non-significant.

Intestinal disease is a characteristic of CF mice, including this strain^21^,^22^,^23^. To determine whether streptomycin treatment affected the CF intestinal disease phenotypes, we measured crypt to villus axis (CVA) height, goblet cell number and muscle thickness in samples procured from both streptomycin treated and untreated mice. The significant increases in CVA height, goblet cell number and muscle thickness of BALB/c *Cftr*^tm1UNC^ mice, compared to wild-type mice, (*P* < 0.002) were not affected by streptomycin treatment (*P* > 0.4; Fig. 2).

**Figure 3:**
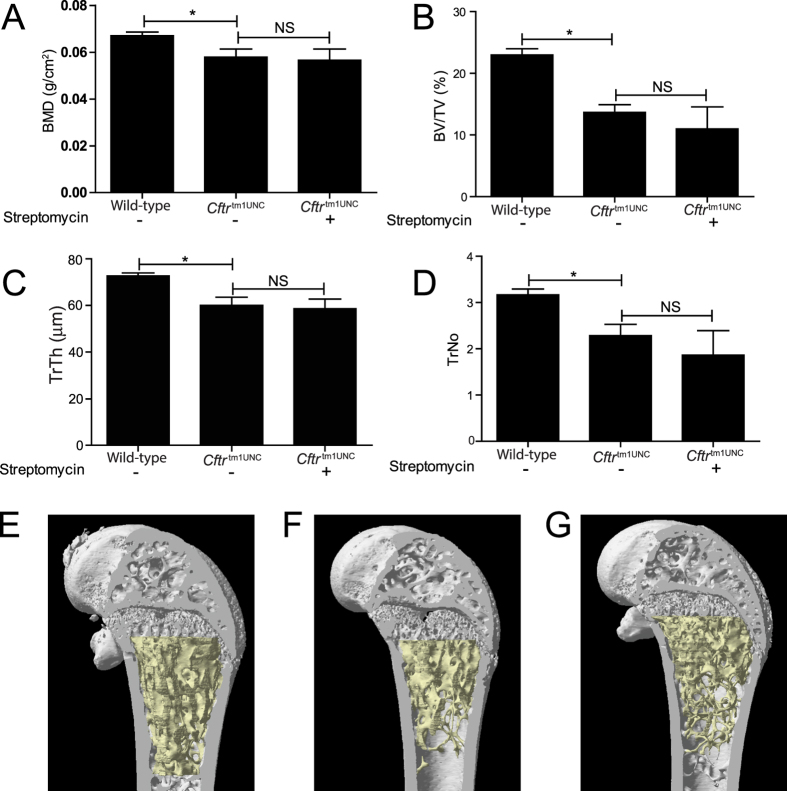
Left femur architecture of female BALB/c *Cftr*^tm1UNC^ mice untreated or treated with Streptomycin *in utero* until death at 12 weeks of age, and of wild-type littermates. (**A**) Bone mineral density (BMD) (**B**) Bone volume to tissue volume (BV/TV) (**C**) Thickness of individual trabeculae (TrTh) (**D**) Number of trabeculae in a given area (TrNo). Average ± standard deviation (n = 4–5 mice per group). Bones from (**E**) WT untreated, (**F**) *Cftr*^tm1UNC^ untreated and (**G**) *Cftr*^tm1UNC^ streptomycin treated mice were dissected free of soft tissue, fixed and scanned on a SkyScan 1072 with three-dimensional reconstruction showing fewer, thinner trabeculae and bone volume in the *Cftr*^tm1UNC^ mice compared to WT mice. *indicates a significant difference between groups, *P* < 0.05, by Student’s *t-*test. NS = non-significant.

Secondly, BALB/c *Cftr*^tm1UNC^ mice have been documented to have a phenotype in femoral tissue that includes reductions in bone mineral density, bone volume to tissue volume, and in trabecular thickness and number^19^. We used Micro-CT to measure bone morphology and although the existence of the CF bone trait was verified, the bone architecture in streptomycin treated *Cftr*^tm1UNC^ mice did not significantly differ from that of untreated *Cftr*^tm1UNC^ mice (*P* > 0.16; Fig. 3).

### Streptomycin treatment ameliorated airway hyperresponsiveness in BALB/c *Cftr* ^*tm1UNC*^ mice

**Figure 4:**
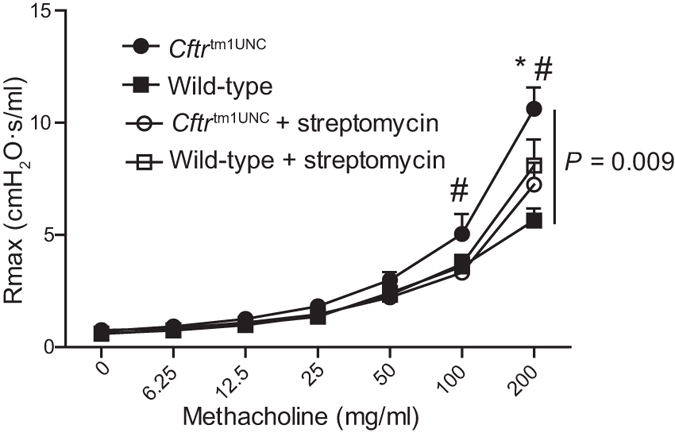
Airway hyperresponsiveness of female BALB/c *Cftr*^tm1UNC^ mice and wild-type littermates, untreated and treated with streptomycin beginning *in utero* until death at 12 weeks of age. Tracheostimized mice received saline (zero) and increasing doses of aeronebulized methacholine and mechanics were assayed on a FlexiVent instrument. Average Rmax, defined as maximal resistance at each dose, ± SEM is shown (n = 9–12 mice per group). Vertical bar indicates a significant difference among groups as measured by repeated measures ANOVA. *indicates a significant difference between untreated *Cftr*^tm1UNC^ mice and untreated WT mice by Bonferroni post hoc test, *P* < 0.05. # indicates a significant difference between untreated *Cftr*^tm1UNC^ mice and streptomycin treated *Cftr*^tm1UNC^ mice by Bonferroni post hoc test, *P* < 0.05.

To investigate whether streptomycin treatment influenced airway disease, we measured the airway response to methacholine challenge of the 12 week old mice. In agreement with a prior report^17^, untreated BALB/c *Cftr*^tm1UNC^ mice displayed an increased airway response to challenge compared to the response of untreated wild-type mice (Fig. 4, *P* = 0.009). Streptomycin treatment significantly reduced the airway hyperresponsive phenotype of *Cftr*^tm1UNC^ mice such that their responses did not differ from those of wild-type mice, (*P* > 0. 05) as shown in Fig. 4.

**Figure 5:**
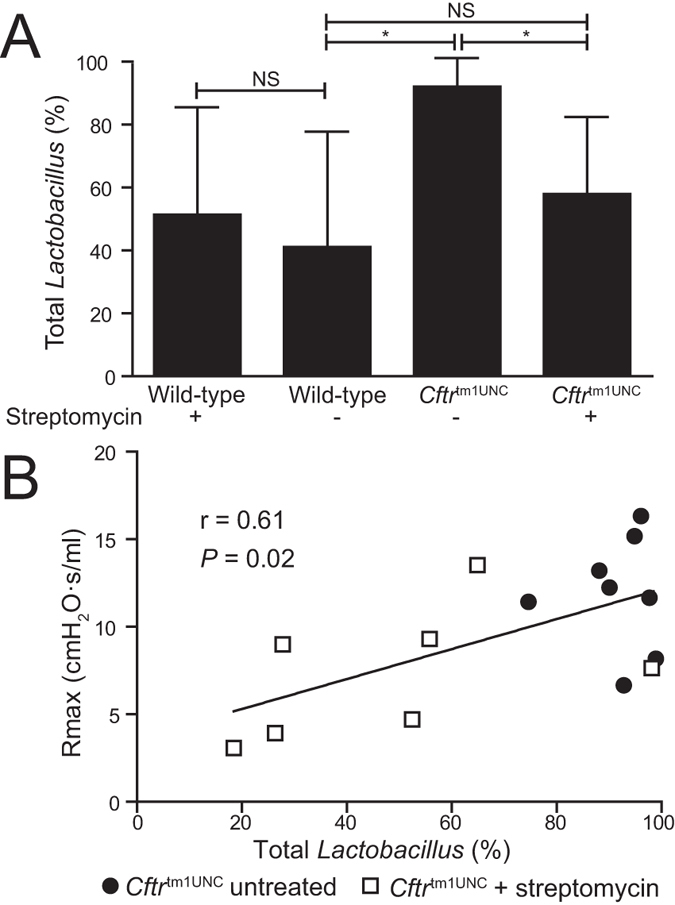
Correlation of airway hyperresponsiveness to total *Lactobacillus* levels. (**A**) Abundance of total *Lactobacillus* within the small intestinal microbiome, based on sequences grouped by taxonomical assignment. Average ± standard deviation is shown (n = 5–13 mice per group). *indicates a significant difference between groups, *P* < 0.05, by Student’s *t-*test. (**B**) Correlation of total *Lactobacillus* abundance to airway hyperresponsiveness in streptomycin treated and untreated *Cftr*^tm1UNC^ mice. Pearson coefficient is shown.

To determine whether the reduced airway response of streptomycin treated BALB/c *Cftr*^tm1UNC^ mice could be related to the treatment-affected intestinal bacterial load or its microbiome constituents, correlation analyses were completed. From these analyses, intestinal bacterial load was identified to be suggestively correlated to an increased airway resistance response (*r* = 0.54, *P* = 0.07) in the combined set of streptomycin treated and untreated *Cftr*^tm1UNC^ mice (data not shown). Next, to identify specific bacteria that may influence this lung phenotype, we reviewed the OTUs that differed significantly in abundance between intestinal samples from *Cftr*^tm1UNC^ mice compared to wild-type mice, in the untreated condition. Of those, we identified the subset for which abundance was also significantly changed by streptomycin treatment in *Cftr*^tm1UNC^ mice, i.e. the CF microbiome features affected by antibiotic treatment. These analyses revealed OTU2, OTU6, and total *Lactobacillus,* the grouping of all sequences classified as *Lactobacillus*, combined, to meet our criteria. As shown in Fig. 5A, total *Lactobacillus* was of significantly increased abundance in untreated *Cftr*^tm1UNC^ mice compared to untreated wild-type mice, and streptomycin treatment reduced *Lactobacillus* in *Cftr*^tm1UNC^ mice to wild-type levels. While intestinal levels of OTU2 or OTU6 did not correlate with airway hyperresponsiveness in *Cftr*^tm1UNC^ mice (*P* > 0.46), analyses revealed the abundance of total intestinal *Lactobacillus* to positively correlate (*r* = 0.61, *P* = 0.02) with airway hyperresponsiveness in *Cftr*^tm1UNC^ mice (Fig. 5B).

### Streptomycin treatment altered the immune profile in the lungs and mesenteric lymph nodes of BALB/c *Cftr* ^tm1UNC^ and wild-type mice

As the immune profile of the lung has been implicated in the development of airway hyperresponsiveness^44^ and given that the intestinal microbiome has been shown to affect the respiratory immune cell populations in other disease models^30^,^33^,^34^, we next identified components of the immune system that may have contributed to the streptomycin ameliorated airway hyperresponsive phenotype in BALB/c *Cftr*^tm1UNC^ mice. Flow cytometric profiling of immune cells present in the lungs and mesenteric lymph nodes of *Cftr*^tm1UNC^ and wild-type mice, in each of the untreated and streptomycin treated conditions, was completed. Lymphocyte subset profiling of the lungs and mesenteric lymph nodes was completed following *ex vivo* PMA/ionomycin stimulation of mixed cell fractions from each tissue.

**Figure 6:**
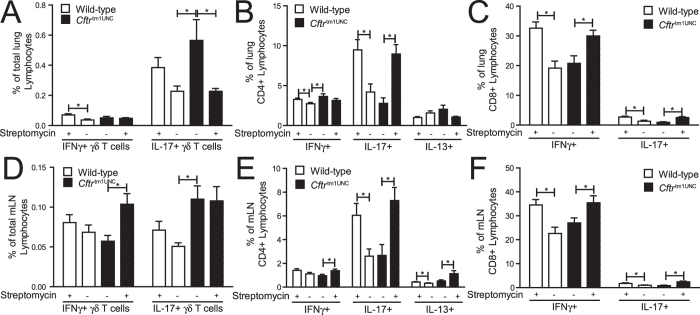
T lymphocyte subsets in the lungs and mesenteric lymph nodes of female BALB/c *Cftr*^tm1UNC^ mice and wild-type littermates, untreated or treated with streptomycin beginning *in utero* until death at 12 weeks of age, as determined by flow cytometry. Specific cytokine producing γδ T cells as a percent of total (**A**) lung or (**D**) mesenteric lymph node lymphocytes. Specific cytokine producing cells as a percent of total (**B**) lung or (**E**) mesenteric lymph node CD4+ lymphocytes; and as a percent of total (**C**) lung or (**F**) mesenteric lymph node CD8+ lymphocytes. IL13 producing γδ T cells and IL13 producing CD8+ lymphocytes were below detection levels in the lungs and mesenteric lymph nodes. Average ± standard deviation is shown (n = 8–14 mice per group). *indicates a significant difference between groups, *P* < 0.05, by Student’s *t-*test. NS = non-significant.

Streptomycin treatment did not affect the numbers of lymphocytes in the lungs of *Cftr*^tm1UNC^ or wild-type mice. As shown in Supplemental Fig S3A, the total number of CD3+, CD4+, and CD8+ lymphocytes, and γδ T cells (CD3 + γδTCR+), did not differ among mice grouped by *Cftr* genotype or by streptomycin treatment. Among T lymphocyte subsets, however, a streptomycin treatment effect on *Cftr*^tm1UNC^ mice was evident as an increase in the percent of IL-17 producing γδ T cells unique to *Cftr*^tm1UNC^ mice (*P* = 0.042 vs. levels in wild-type mice), was detected as presented in Fig. 6A, and streptomycin treatment reduced the percent of these cells in *Cftr*^tm1UNC^ mice (*P* = 0.041) to wild-type levels. An increased percentage of Th1 (CD4 + IFNγ+) lymphocytes was also detected in the lungs of *Cftr*^tm1UNC^ mice (*P* = 0.031 vs. levels in wild-type mice), as presented in Fig. 6B, but streptomycin treatment did not significantly affect this cell population in *Cftr*^tm1UNC^ mice (*P* = 0.25). A streptomycin influence on pulmonary lymphocyte populations which affected both *Cftr*^tm1UNC^ and wild-type mice was also revealed, as depicted in Fig. 6B,C wherein increased percentages of Th17 (CD4 + IL-17+) lymphocytes, CD8+ IFNγ+ lymphocytes and CD8+ IL-17+ lymphocytes were evident in treated, compared to untreated, mice irrespective of *Cftr* genotype. The expression levels of *Il13*, *Il5*, and *Ifnγ*, in the whole lung, did not differ among mice grouped by *Cftr* genotype or streptomycin treatment (P > 0.32) and *Il17* expression was below the detection level (Supplemental Figure S4.)

The CF status of the mice and streptomycin treatment produced limited effects on the innate immunity cell profile of the lungs, as shown in Supplemental Fig S5. Specifically, a streptomycin increase in alveolar macrophages (CD45 + CD11c + CD11b-CD64 + CD24-) was evident in both *Cftr*^tm1UNC^ and wild-type mice while eosinophils were increased in *Cftr*^tm1UNC^ mice (*P* < 0.0005) to wild-type levels (*P* = 0.10), after streptomycin treatment.

To determine whether the streptomycin treatment associated changes in the pulmonary cell profile were reflected in the immune profile of the intestine, we assayed the mesenteric lymph nodes. Streptomycin treatment did not significantly affect the general T lymphocyte profile of *Cftr*^tm1UNC^ mice, as the total numbers of CD3+, CD4+ and CD8+ lymphocytes and γδ T cells did not differ between treated and untreated *Cftr*^tm1UNC^ mice (*P* > 0.10; Supplemental Fig. S3B). As was evident in the lungs, an increase in IL-17 producing γδ T lymphocytes, relative to levels in untreated wild-type mice (*P* = 0.007) was detected in the nodes of *Cftr*^tm1UNC^ mice; as shown in Fig. 6D, although in this tissue streptomycin treatment did not alter the percent of IL-17 producing γδ T cells (*P* = 1.0). In further similarity to its effects on the lung, streptomycin treatment resulted in significantly increased levels of Th17 lymphocytes, CD8 + IL-17+ lymphocytes and CD8+ IFNγ+ lymphocytes, in the mesenteric lymph nodes of both *Cftr*^tm1UNC^ and wild-type mice (Fig. 6E,F).

Overall, these results indicate that streptomycin treatment decreased the CF phenotype of increased pulmonary IL-17 producing γδ T lymphocytes while causing an increase in Th17, CD8 + IL-17+ and CD8 + IFNγ+ lymphocytes in both the lung and mesenteric lymph nodes.

## Discussion

In this work, we show streptomycin treatment decreased the intestinal bacterial overgrowth in BALB/c *Cftr*^tm1UNC^ mice and affected the microbiome, corresponding with changes to the pulmonary lymphocyte profile and a decreased airway response.

Our findings reflect emerging clinical data and suggest an intestinal microbiome influence on lung disease in cystic fibrosis. Specifically, in support of such an influence, Hoen *et al.*^45^ recently reported the intestinal, but not respiratory, microbiome profile to associate with respiratory exacerbation in CF children. Further, studies have shown the use of probiotics, an intervention which affects the intestinal microbiome^9^, to reduce the number of pulmonary exacerbations^46^,^47^,^48^ and, in a pilot study, to increase the lung function^46^ of CF patients. Secondly, the altered lymphocyte profile of the BALB/c *Cftr*^tm1UNC^ mice, with respect to wild-type mice, replicates clinical observations where aggregates of pulmonary T cells have been described^49^,^50^,^51^, and may affect patient susceptibility to infection^52^,^53^. In mice, changes in the pulmonary lymphocyte profile likely affected CF related airway hyperresponsiveness^4^, as has been established in models of asthma^44^, but whether or how the altered immune profile of the *Cftr*^tm1UNC^ mice affected susceptibility to infection was not assessed in the present work as all mice were housed in specific pathogen free conditions.

In streptomycin treated BALB/c *Cftr*^tm1UNC^ mice the reduction in airway hyperresponsiveness coincided with CF-specific and streptomycin mediated changes in the adaptive immune response. Firstly, antibiotic treatment reduced the increase in pulmonary IL-17 producing γδ T cells of *Cftr*^tm1UNC^ mice, an immune response to antibiotics which has been reported in a lung cancer model^54^, and this reduction in γδ T cells may have affected the airway response as Matsubara *et al.*^55^ have shown γδ T cells to be required for hyperresponsiveness to ozone challenge in mice. Others have reported γδ T cells to be protective against this trait in experimental asthma^56^,^57^, in contrast to our findings, although the γδ T cell contribution to lung disease has also been shown to depend on the immune environment in which they act^58^. To this point, although an effect on IL-17 producing γδ T cell number was measured in streptomycin treated *Cftr*^tm1UNC^ mice, we did not detect an effect on expression of interleukin-17, at the level of the whole lung, in these mice compared to controls, and therefore further testing will be required to determine whether streptomycin affects airway hyperresponsiveness in this model via modulation of IL-17 producing γδ T cells. If confirmed, the increase in pulmonary IL-17 producing γδ T cells of *Cftr*^tm1UNC^ mice may also be clinically significant as increased levels of IL-17 have been documented in the lungs of CF patients^52^,^59^,^60^,^61^ and have been correlated to lung tomographic changes in this group^52^.

Secondly, streptomycin treatment also affected the levels of specific lymphocytes in *Cftr*^tm1UNC^ and wild-type mice, similarly, which is consistent with reported microbiome effects on immunity both locally^29^,^62^ and in the lung^30^,^33^,^34^,^36^. The lymphocyte profile of BALB/c *Cftr*^tm1UNC^ mice, therefore occurred in a pulmonary immune environment which was altered as a result of streptomycin treatment, and this effect itself may have lessened the airway response in *Cftr*^tm1UNC^ mice, based on data from related models. For example, Russell *et al.*^39^ reported that streptomycin treatment, which produced a changed intestinal microbiome, also affected the severity of hypersensitivity pneumonitis in the lungs of treated mice, through an adaptive immunity mediated response. Further, work of Noverr *et al.*^63^ showed antibiotic-induced gut microbiome perturbation to drive the development of a T-cell-mediated airway response in BALB/c mice without requiring previous systemic antigen priming, a phenomenon which is similar to the response of BALB/c *Cftr*^tm1UNC^ mice reported here.

Streptomycin’s effect on airway hyperresponsiveness in BALB/c *Cftr*^*tm1UNC*^ mice may have been mediated through levels of *Lactobacillus* in the intestine. In detail, the most abundant bacteria in the intestines of BALB/c *Cftr*^*tm1UNC*^ mice were *Lactobacillus*, which were detected at significantly greater levels than in the intestines of their wild-type littermates. The increased abundance of *Lactobacillus* in the *Cftr*^tm1UNC^ mice may have resulted from its capacity to grow in the lower pH environment of the CF intestine^64^. Streptomycin treatment depleted the intestinal *Lactobacillus*, as has been reported by others^65^, resulting in a range of intestinal *Lactobacillus* levels in BALB/c *Cftr*^*tm1UNC*^ mice which positively correlated with increased airway hyperresponsiveness. Similarly, treatment of mice with a different antibiotic, vancomycin, was reported to increase both the airway hyperresponsiveness of ovalbumin challenged mice and the abundance of *Lactobacillus* in their fecal samples^32^. While the mechanism affecting the airway response was not elucidated the authors speculated that the increased level of *Lactobacillus*, normally a small component of the intestinal microbiome, may be harmful. Importantly, *Lactobacillus* has immunomodulatory effects in other disease models^66^,^67^. Whether the *Lactobacillus* overgrowth alone, or in the context of the CF environment, with or without other bacteria, affected the CF airway or immune responses, requires further investigation. Finally, although this antibiotic is reportedly poorly absorbed^41^ we can not rule out the possibilities that streptomycin treatment may have affected the pulmonary microbiome, or may have produced a non-antibiotic related effect on inflammation in the mice^68^, and for either of these influences to have, in turn, altered the pulmonary traits of the mice.

The microbial changes induced by streptomycin did not produce measureable effects on CF bone, intestinal disease or body weight, suggesting these traits are either not microbiome driven in BALB/c *Cftr*^tm1UNC^ mice, or that more profound or directed antibiotic intervention than that investigated here is required to produce an effect. Specifically, in this work, we showed the BALB/c *Cftr*^tm1UNC^ mice to have altered bone architecture and reduced bone mineral density, in agreement with a prior study^19^, but that these phenotypes were not affected by streptomycin treatment. Given that Sjogren *et al.*^35^ detected an increase in bone mineral density in germfree wild-type mice, the lack of an effect on the bone disease in the *Cftr*^tm1UNC^ mice may have occurred as a result of streptomycin treatment reducing, but not eliminating, intestinal bacteria. Similarly, streptomycin treatment did not influence the body weight of *Cftr*^tm1UNC^ mice in this work, although high dose treatments of ciprofloxacin and metronidazole were reported to increase body weight in C57BL/6 *Cftr*^tm1UNC^ mice^27^ indicating an antibiotic dose or specificity contribution to this trait. Finally, the CF-associated changes in the intestine^21^,^22^,^23^, similarly replicated here, were also not measurably affected by the reduced bacterial load or by an altered microbiome. For the trait of circular muscle thickness, this is in contrast to our previous findings^21^; wherein the thickness was positively correlated with increasing intestinal bacterial load in the combined dataset of three separate lines of Cftr deficient mice. The absence of an effect measured here indicates these traits may be influenced by specific bacteria increased in *Cftr*^tm1UNC^ mice that are still present post streptomycin treatment, or by factors that are independent of the intestinal microbiome.

In this study, we demonstrate that altering the intestinal microbiome and bacterial load in BALB/c *Cftr*^tm1UNC^ mice with streptomycin treatment were associated with reduced airway hyperresponsiveness, while intestinal and bone disease were not affected by the components of the microbiome influenced by streptomycin. Further, an altered immune system profile, including increases in the percent of Th17, CD8 + IL-17+ and CD8 + IFNγ+ lymphocytes, and a reduction in the CF lung phenotype of an augmented percent of IL-17 producing γδ T cells culminated to associate with the decreased CF airway response. This is therefore the first study to demonstrate that the intestinal microbiome may influence airway mechanics in CF, thus providing a novel pathway through which mechanistic insight into CF lung disease may be gained.

## Materials and Methods

### Mice

BALB/c *Cftr*^+/tm1UNC^ heterozygous mice were used to generate *Cftr*^tm1UNC^ mice as previously described^17^,^69^. Mice were genotyped^69^ and maintained at the Meakins-Christie Laboratories at McGill University. To prevent potential premature death due to intestinal disease, all mice were fed standard chow and received PEGLYTE® (17.8 mmol/L polyethylene glycol, Pharma Science, Montreal, Canada) in their drinking water as described previously^17^,^69^,^70^. Female mice were weaned at 3 weeks of age and grouped in ventilated cages. Within treatment groups, *Cftr*^tm1UNC^ and wild-type mice were cohoused where possible. To reduce the intestinal bacterial load and to alter the microbiome, the antibiotic streptomycin was used. Streptomycin is an aminoglycoside group antibiotic that has antibacterial activity against both gram-negative and gram-positive bacteria, and, was selected for this investigation based on reports that is not absorbed in the intestine^32^,^41^. Principally because of its pharmokinetic profile, streptomycin is not used in clinical CF, where treatment with other aminoglycoside group antibiotics is favoured^71^. For the streptomycin treated groups, experimental mice and their direct progenitors were maintained on streptomycin (200mg/L; Sigma, St. Louis, MO) in their drinking water. The drinking water was changed twice weekly and water consumption was not recorded. Mice were thus treated from *in utero* until sacrifice at 12 weeks of age. Mice were weighed weekly from 5 to 12 weeks of age. At this time airway mechanics were measured before completion of euthanasia by cardiac puncture and subsequent tissue collection. Additional mice were anaesthetized and euthanized by cardiac puncture before tissue collection. After euthanasia, the small intestine was removed and the contents collected by flushing with phosphate buffered saline containing 10mM dithiothreitol as a mucolytic agent. The tissue was fixed in formalin and submitted for standard histological processing. The left femur was collected and stored in phosphate buffered saline before being analyzed. For a second cohort of mice, lungs and mesenteric lymph nodes (mLN) were excised for flow cytometric analysis subsequent to euthanasia. All animal procedures were performed under protocol 4653 which was approved by the McGill University Animal Care Committee. The regulations for the McGill committee adhere to those set by the Canadian Council on Animal Care.

### Bacterial load measurement

The small intestinal contents were pelleted through centrifugation at 19000 rpm for 30 minutes. Intestinal bacterial DNA was extracted from 150 mg of the centrifuged small intestinal contents using a Stool DNA Kit (Qiagen, Venlo, Netherlands). Bacterial load was quantified by real-time PCR amplification of the 16S rRNA gene as previously described^21^,^23^,^72^. A standard curve of the number of 16S rRNA copies was created by extracting and quantifying DNA from a known number of *Escherichia coli*.

### Bacterial DNA extraction and PCR amplification of the 16S rRNA gene

Bacterial DNA from the small intestinal contents was extracted using a repeated bead beating and column extraction method^10^,^21^,^73^. A survey of the microbiome was completed using FLX Pyrosequencing of the V4-V6 region of the 16S rRNA gene by MrDNA (Shallowater, TX) as previously described^10^,^74^ (Primers: 530-F: GTGCCAGCMGCNGCGG and 1100-R: GGGTTNCGNTCGTTG). 10000 reads per sample were requested. Raw data were analyzed using Mothur version 1.32^75^ and cleaned and aligned as previously described^10^,^76^. Reads were binned into Operational Taxonomic Units (OTU) based on 97% sequence similarity and the most abundant read for each OTU was selected as the representative read for purposes of taxonomical assignment with the Ribosomal Database Project classifier^77^. To assess the abundance of taxonomical groups within a sample, reads were binned based on taxonomical assignment at the phylum level. The dataset was deposited into the NCBI Sequence Read Archive (accession number PRJNA288223).

### Histology

Histological structures were evaluated on formalin-fixed paraffin embedded small intestinal sections (5 μm) stained with haematoxylin and eosin. The villus height, crypt depth and total crypt to villus axis (CVA) height were measured on 25 complete and intact CVAs within each ileum, using image analysis of the histological sections (Olympus BX51, Image-Pro Plus 5.1, Media Cybernetics, Rockville, MD) as in previous studies^21^,^22^,^23^. Goblet cell numbers were counted on 25 ileal CVAs per mouse using Periodic acid-Schiff/Alcian blue stained sections. The circular and longitudinal thickness of the muscularis externa layer was measured at 50 positions located at regular intervals throughout the ileum. All sections were scored by an observer blinded to *Cftr* genotype and treatment.

### Airway mechanics

The airway response to methacholine challenge was measured as described^17^,^18^. After being anaesthetized with intraperitoneal injections of xylazine hydrochloride (11.3 mg/kg) and sodium pentobarbital (37 mg/kg), mice were paralyzed with pancuronium bromide (0.2 mg). A tracheostomy tube was inserted into the exposed trachea and connected to a computer-controlled ventilator (FlexiVent; SCIREQ®). The ventilator parameters were set at 150 breaths/min, tidal volume of 10 mL/kg, and a positive end-expiratory pressure (PEEP) of approximately 3.0 cmH_2_O. Resistance measurements were recorded using the Aeroneb ultrasonic nebulizer (SCIREQ®) on the FlexiVent system in response to baseline aerosol saline administration and subsequently to doubling doses of methacholine (6.25 – 200 mg/mL).

### Femur radiological imaging

Bone phenotyping procedures were performed as previously described^19^,^78^. Bone mineral density was measured on a PixiMUS densitometer (Lunar, GE-Healthcare Madison, WI, USA) and bone morphometry assessed using a Skyscan 1072 Micro-CT instrument (Skyscan, Aartselaar, Belgium). Micro-CT imaging was performed at settings of 45 kV/222 μA; spatial resolution of 5.63 μm/pixel; 2.2 second exposure per frame and 0.9 degree rotation between frames. These two-dimensional images were used to reconstruct three-dimensional images for analysis using CT-Analyzer software (v 1.10.02). 2.26 mm of trabecular bone, starting from the growth plate, was analysed.

### Flow cytometry of lungs and mesenteric lymph nodes

At necropsy, lungs and mesenteric lymph nodes (mLN) were removed. Lungs were cut into small pieces and placed in PBS containing 0.5% BSA, 1 mg/mL collagenase (Roche) and 0.1 mg/mL DNAse (Roche). Lung tissue was further disrupted using a Cell Dissociation Kit (Sigma-Aldrich) as per manufacturer’s directions. Red blood cells were lysed with red blood cell lysis buffer (BioLegend). Mesenteric lymph nodes were disrupted by grinding the tissue through a 70 μm strainer. For detection of dendritic cells, macrophages and granulocytes, cells were stained with anti-CD45 (eBioscience, Pe-Cy5.5, clone 30-F11), CD11b (eBioscience, APC, clone M1/70), CD11c (BioLegend, Pe-Cy7, clone N418), CD64 (BioLegend, BV421, X54-5/7.1), CD24 (BioLegend, PE, clone M1/69), Siglec F (BD Bioscience, PE-CF594, clone E50-2440), and MHC Class II (BD Bioscience, V500, M5/114.15.2). Cells were acquired on an LSR II instrument (BD Bioscience) and analyzed with FlowJo software (FlowJo, LLC) using the gating strategy of Misharin *et al.*^79^, shown in Supplemental Fig S6A. Cells were defined as CD11b+ dendritic cells, CD45 + CD11c + CD11b + CD24 + CD64-MHC class II+; alveolar macrophages CD45 + CD11c + CD11b-CD64 + CD24-; interstitial macrophages, CD45 + CD11c + CD11b + CD24-CD64 + MHC class II+; eosinophils, CD45+ CD11b + MHC class II-CD24 + Siglec F+; and neutrophils, CD45+ CD11b + MHC class II-CD24 + Siglec F-.

For detection of T lymphocytes subsets, lung and mLN cells were stimulated in complete media with 50 ng/mL PMA (Sigma-Aldrich), 1 μg/mL ionomycin (Sigma-Aldrich), and 1 μl/mL Golgi Stop (BD Bioscience) for 4 hours at 37 °C. After stimulation, cells were stained for the extracellular markers of viability (eBioscience), CD3 (eBioscience, FITC, clone 145-2C11), CD4 (eBioscience, APC, clone GK1.5), CD8 (eBioscience, Pe-Cy5.5, clone 53-6.7) and γδ TCR (BD Bioscience, PE, clone GL3). Cells were then fixed and permeabilized with Cytofix/Cytoperm (BD Bioscience) and Cytoperm Plus Buffer (BD Bioscience). After permeabilization, cells were stained with antibodies for IL-13 (eBioscience, Pe-Cy7, clone ebio13A), IL-17A (BD Bioscience, BV510, clone TC11-18H10), IFNγ (BD Bioscience, PE-CF594, clone XMG1.2). Cells were acquired on an LSR II instrument (BD Bioscience) and analyzed with FlowJo software (FlowJo, LLC) using a gating strategy summarized in Supplemental Fig 6B.

### Quantitative real-time PCR

Gene expression experiments were completed as previously described^22^. Briefly, total RNA was isolated from the right mouse lung and reverse transcribed with oligo(dT) primers using Superscript III RNase H-Reverse Transcriptase (Invitrogen). Quantitative real-time PCR was performed on this cDNA using the Applied Biosystems International Prism 7500 Sequence Detection instrument and Taqman (ThermoFisher Scientific) assays for *Il-17A* (Interleukin 17A, Mm00439619_m1), *Ifnγ* (Interferon gamma, Mn01168134_m1), *Il-5* (Interleukin 5, Mn00439646_m1) and *Il-13* (Interleukin 13, Mm00434204_m1). Ataxin 10 (Mm00450332_m1) was used as the reference gene. Relative expression was calculated using the comparative C_T_ method and significance was evaluated with Student’s t test.

## Statistical Analysis

Weight, histological, bacterial load, immune profile, and bone phenotypes are expressed as the mean ± standard deviation of mice grouped by *Cftr* genotype and treatment. Significant differences in the mean between groups were determined using Student’s *t-*test, and a significance threshold of *P* < 0.05. Airway hyperresponsiveness to methacholine was analyzed using a repeated measure ANOVA followed by a Bonferroni post-test. Correlations among phenotypes were evaluated using Pearson’s correlation coefficient. Differences in survival rates at either weaning or sacrifice, between mice grouped by *Cftr* genotype and treatment, were determined by Fisher’s exact test in Graphpad Prism V5.03.

With the microbiome sequence dataset, phylogenetic trees were constructed using Clearcut^80^ via Mothur. To investigate the compositional similarity between samples, Bray-Curtis dissimilarities were calculated for each model and were visualized using two dimensional non-metric dimensional scaling (NMDS) ordination. Diversity was measured using Shannon’s Diversity as implemented in Mothur^81^. Differences in abundance of OTUs between mice grouped by *Cftr* genotype and treatment were determined by the Mothur implementation of the Metastats program^82^ with *P* < 0.05 taken as the level of significance.

## Additional Information

**How to cite this article**: Bazett, M. *et al.* Streptomycin treatment alters the intestinal microbiome, pulmonary T cell profile and airway hyperresponsiveness in a cystic fibrosis mouse model. *Sci. Rep.* **6**, 19189; doi: 10.1038/srep19189 (2016).

## Supplementary Material

Supplemental Figures

## Figures and Tables

**Table 1:** Intestinal Microbiome Operational Taxonomic Units (OTU) differing in abundance between BALB/c *Cftr* ^tm1UNC^ mice and wild-type (WT) littermates.

OTU number	Taxonomic assignment*	WT untreated average ± SEM	CF untreated average ± SEM	*P-*value
OTU 1	*Lactobacillus*	6.22^#^ ± 3.45	29.07 ± 5.03	0.0004
OTU 2	*Lactobacillus*	12.08 ± 3.74	28.02 ± 4.03	0.002
OTU 6	*Akkermansia*	8.46 ± 4.34	0.30 ± 0.09	0.036
OTU 11	Porphyromonadaceae	5.93 ± 2.44	0.04 ± 0.03	0.005
OTU 25	*Enterorhabdus*	0.57 ± 0.24	0.05 ± 0.03	0.010
OTU 44	Coriobacteriaceae	0.17 ± 0.06	0.002 ± 0.002	0.003
OTU 46	Coriobacteriaceae	0.13 ± 0.06	0.01 ± 0.005	0.016

^#^abundance in percent.

*highest resolution classification by sequence comparison to the Ribosomal Database Project classifier.

**Table 2:** Intestinal Microbiome Operational Taxonomic Units (OTU) differing in abundance between Streptomycin treated and untreated BALB/c *Cftr* ^tm1UNC^ mice.

OTU number	Taxonomic assignment*	CF untreated average ± SEM	CF treated average ± SEM	*P-*value
OTU 2	*Lactobacillus*	28.02^#^ ± 4.03	4.44 ± 3.59	0.001
OTU 3	Erysipelotrichaceae	1.91 ± 1.25	16.72 ± 4.85	0.003
OTU 5	*Lactobacillus*	24.05 ± 4.00	0.00 ± 0.00	0.001
OTU 6	*Akkermansia*	0.30 ± 0.09	6.71 ± 2.81	0.001
OTU 7	Erysipelotrichaceae	0.09 ± 0.07	0.002 ± 0.002	0.037
OTU 9	*Blautia*	0.52 ± 0.15	2.81 ± 0.75	0.001
OTU 11	Porphyromanadaceae	0.04 ± 0.03	0.00 ± 0.00	0.001
OTU 12	*Anaerostipes*	0.10 ± 0.04	0.91 ± 0.22	0.001
OTU 14	*Stenotrophomanas*	0.03 ± 0.02	1.15 ± 0.44	0.007
OTU 15	*Pseudomonas*	0.02 ± 0.01	1.42 ± 0.77	0.004
OTU 16	*Bidifobacterium*	0.98 ± 0.42	0.002 ± 0.002	0.0001
OTU 22	Enterobacteriaceae	0.70 ± 0.52	0.00 ± 0.00	0.001
OTU 30	*Clostridium_XVIII*	0.05 ± 0.02	0.00 ± 0.00	0.001
OTU 32	*Olsenella*	0.007 ± 0.005	0.13 ± 0.05	0.001
OTU 33	*Coprobacillus*	0.004 ± 0.0003	0.16 ± 0.05	0.003
OTU 37	*Clostridium_XIVa*	0.01 ± 0.01	0.17 ± 0.09	0.019
OTU 41	Coriobacteriaceae	0.00 ± 0.00	0.03 ± 0.02	0.049
OTU 48	*Blautia*	0.02 ± 0.02	0.00 ± 0.00	0.0003
OTU 49	*Staphylococcus*	0.002 ± 0.002	0.08 ± 0.03	0.024

^#^abundance in percent.

*highest resolution classification by sequence comparison to the Ribosomal Database Project classifier.
