# Supplementary material for: Streptomycin treatment alters the intestinal microbiome, pulmonary T cell profile and airway hyperresponsiveness in a cystic fibrosis mouse model
Source: Sci Rep. 2016 Jan 12;6:19189. doi: 10.1038/srep19189 (PMC4709690; doi:10.1038/srep19189)
Supplement: Supplemental Figures [file srep19189-s1.pdf]

# Streptomycin treatment alters the Intestinal Microbiome, Pulmonary T Cell profile and Airway Hyperresponsiveness in a Cystic Fibrosis Mouse Model

**Mark Bazett Marie-Eve Bergeron and Christina K. Haston**

Supporting Information

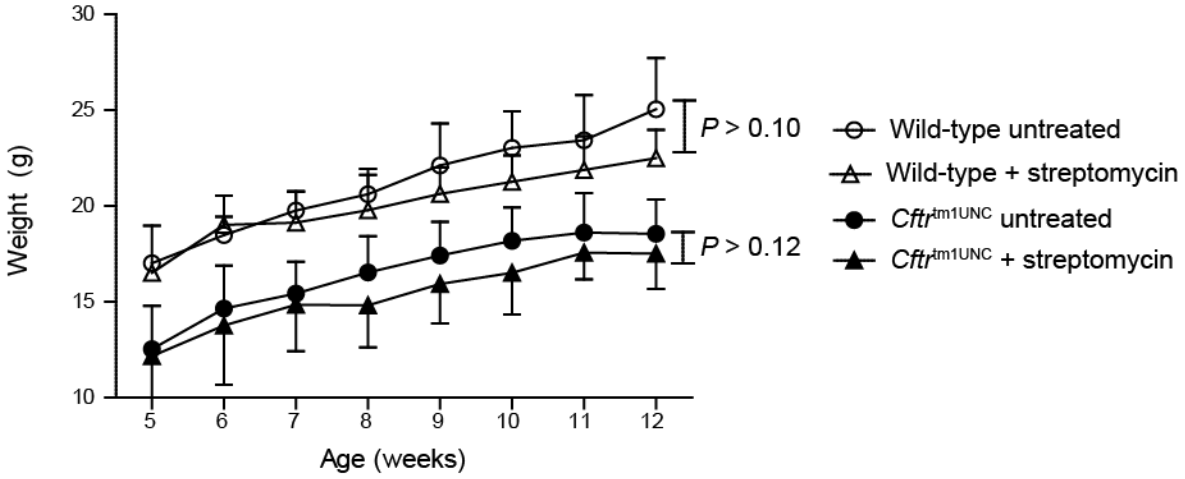


**S1 Figure. Body weight of female BALB/c *Cftr*$^{tm1UNC}$ mice and wild-type littermates, untreated or treated with streptomycin beginning *in utero* until death at 12 weeks of age.** Average weight ± standard deviation of n=6-10 mice per group. Weight did not significantly differ between untreated and Streptomycin treated mice for either of *Cftr*$^{tm1UNC}$ or wild-type mice at any age (Student's *t*-test).

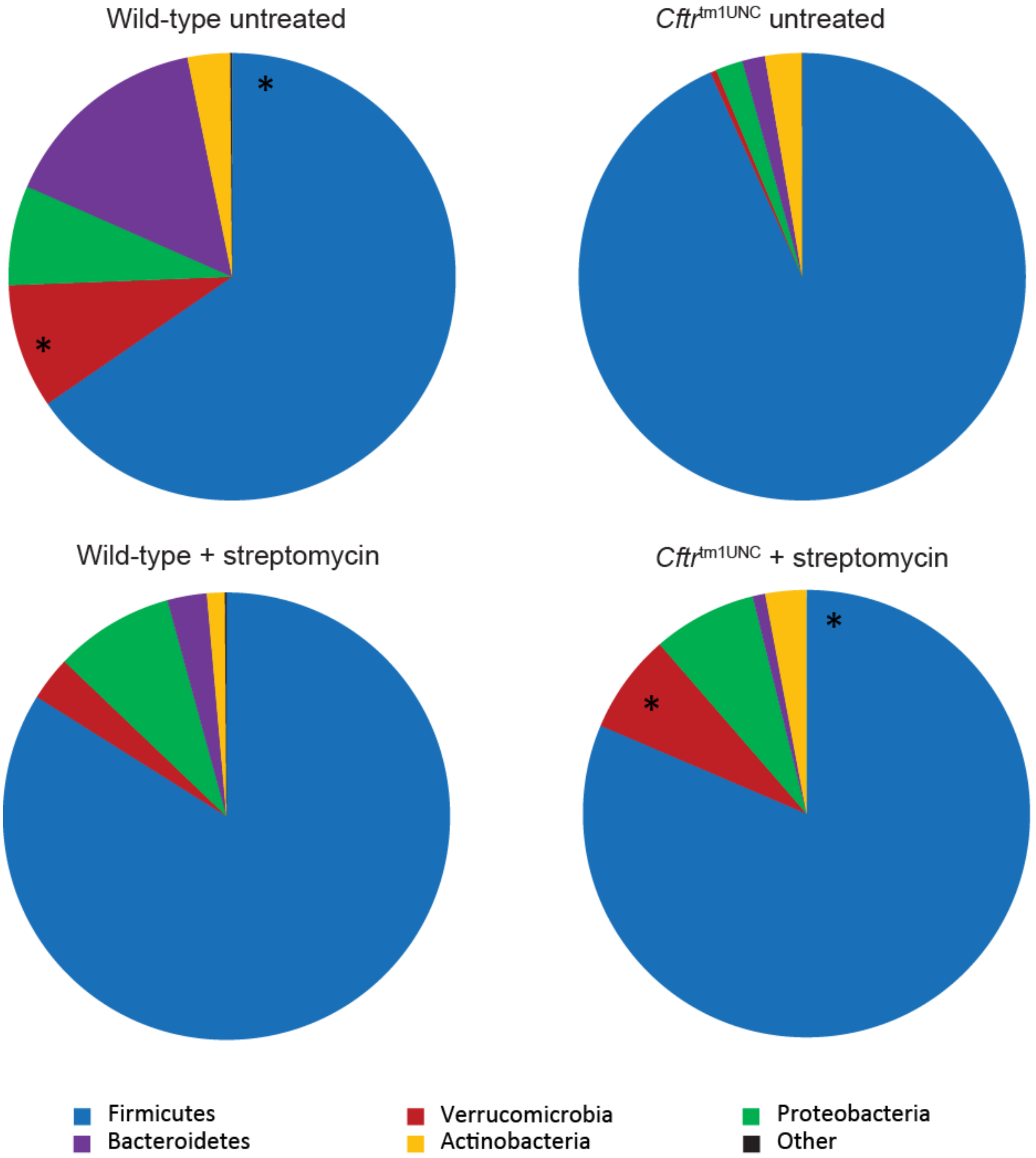


**S2 Figure. Phylum level classification of intestinal microbiome from female BALB/c *Cftr*$^{tm1UNC}$ mice and wild-type littermates, untreated and treated with streptomycin beginning *in utero* until death at 12 weeks of age.** * indicates a significant difference, $P < 0.05$ in bacterial abundance at the phylum level from untreated *Cftr*$^{tm1UNC}$ mice. There was no significance difference in bacterial abundance at the phylum level among untreated wild-type, streptomycin treated wild-type or streptomycin treated *Cftr*$^{tm1UNC}$ mice. The group "other" contains the phylum Tm7, Tenericutes, Acidobacteria, Planctomycetes and unclassified bacteria.

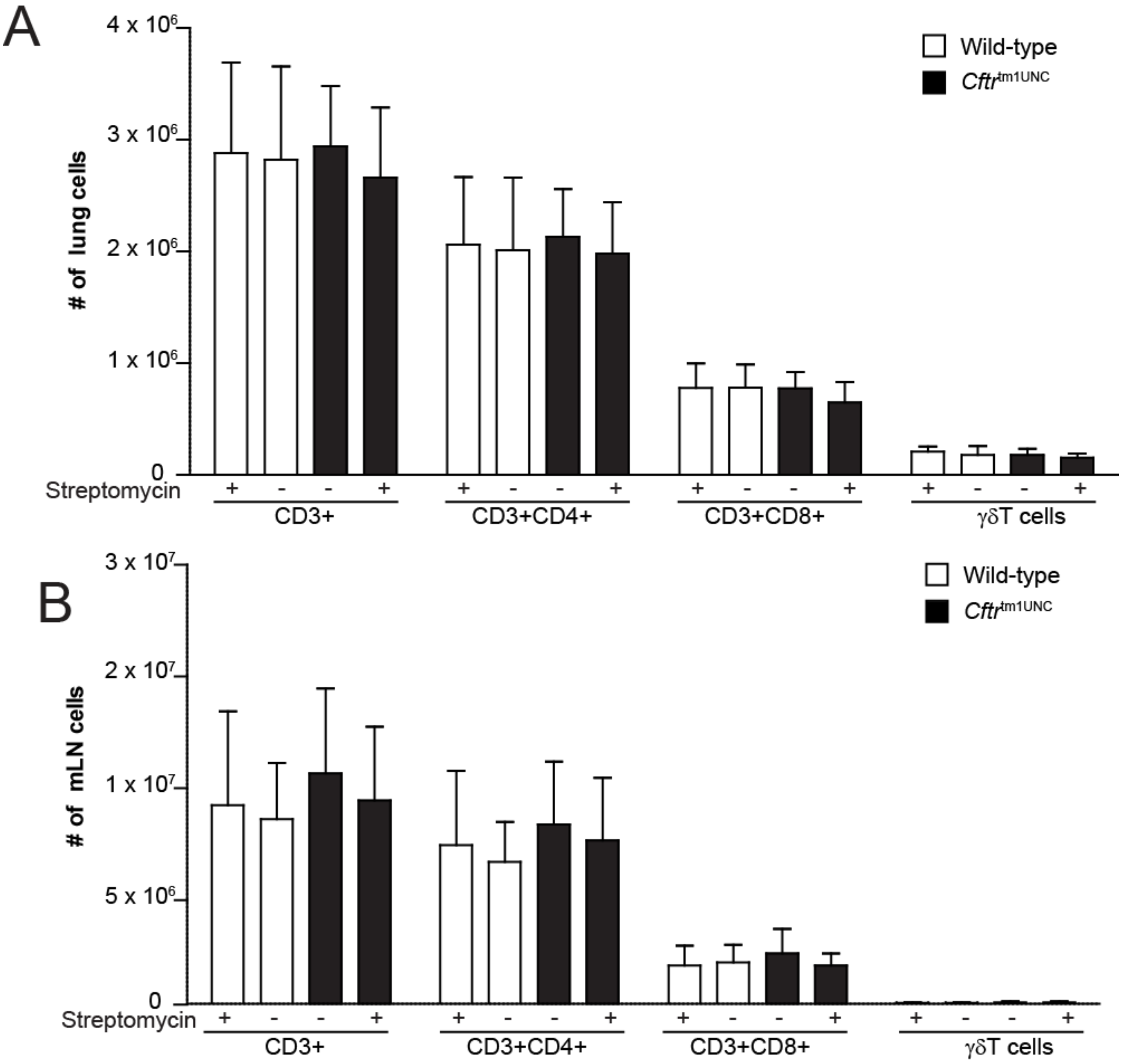


**S3. Figure. Lymphocyte profiling of the lungs and mesenteric lymph nodes of female BALB/c *Cftr*$^{tm1UNC}$ mice and wild-type littermates, untreated or treated with streptomycin beginning *in utero* until death at 12 weeks of age, as determined by flow cytometry.** T lymphocyte subsets in the (A) lungs or (B) mesenteric lymph nodes. Average ± standard deviation is shown (n=6-11 mice per group). * indicates a significant difference between groups, *P* < 0.05, by Student's *t*-test.

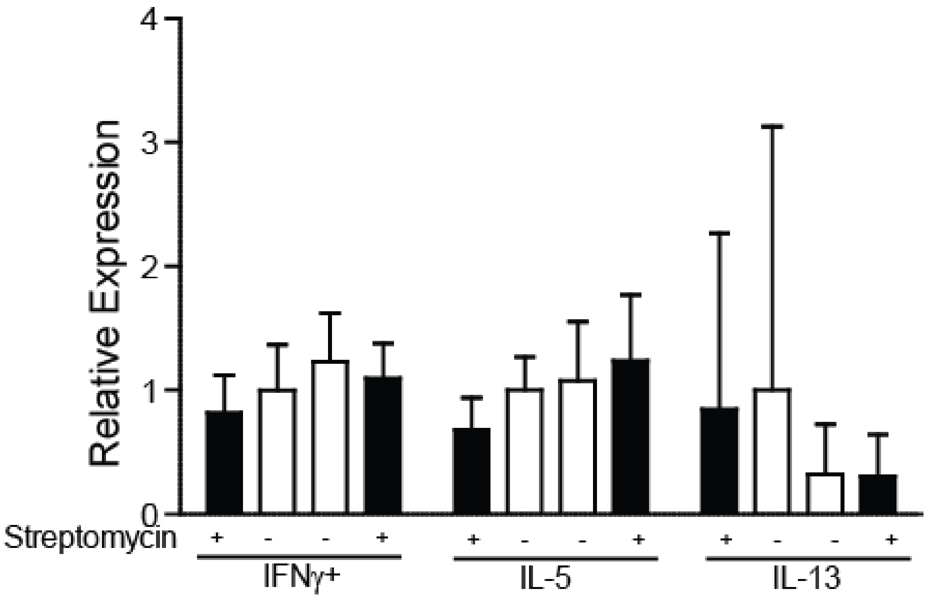


**S4 Figure. Right lung cytokine gene expression of female BALB/c *Cftr*$^{tm1UNC}$ mice and wild-type littermates, untreated or treated with streptomycin beginning *in utero* until death at 12 weeks of age.** Quantitative real-time PCR measures of expression presented as the average ± standard deviation, relative to the reference gene Ataxin 10, of 4-9 mice per group. * indicates a significant difference between groups, $P < 0.05$, by Student's *t*-test. *Il-17A* expression was below detection for most samples.

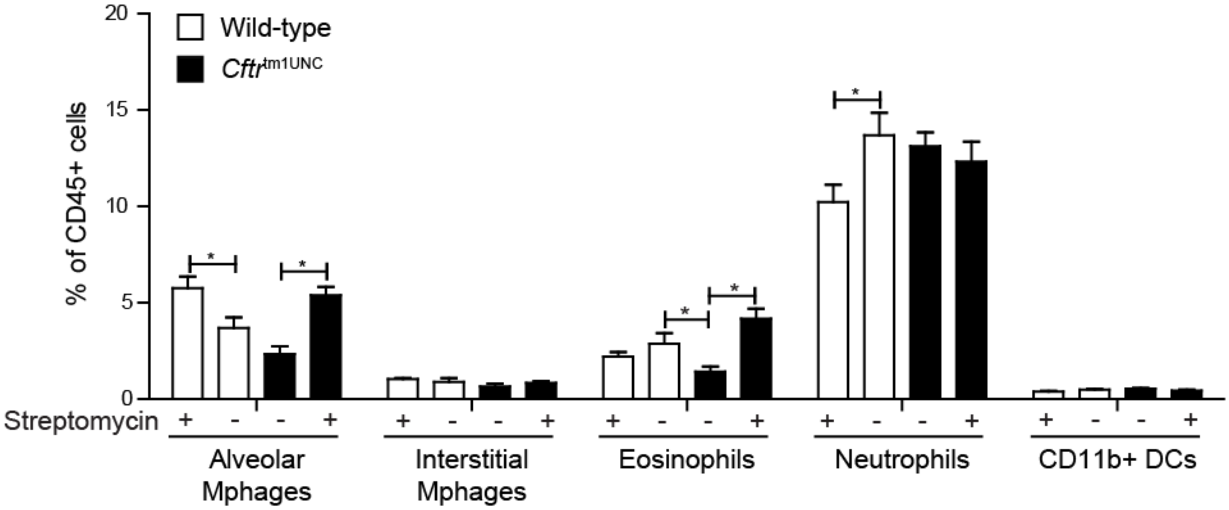


**S5. Figure. Leukocyte profiling of the lungs of female BALB/c *Cftr*$^{\mathbf{tm1UNC}}$ mice and wild-type littermates, untreated or treated with streptomycin beginning *in utero* until death at 12 weeks of age, as determined by flow cytometry.** Alveolar macrophages, interstitial macrophages, eosinophils, neutrophils, and CD11b$^+$ dendritic cells as a percent of lung CD45$^+$ cells. Mphage = macrophages; DC = dendritic cells. CD11b+ dendritic, CD45$^+$CD11c$^+$CD11b$^+$CD24$^+$CD64$^-$MHC class II$^+$; alveolar macrophages CD45$^+$CD11c$^+$CD11b$^-$CD64$^+$CD24$^-$; interstitial macrophages, CD45$^+$CD11c$^+$CD11b$^+$CD24$^-$CD64$^+$MHC class II$^+$; eosinophils, CD45$^+$CD11b$^+$MHC class II$^-$CD24$^+$Siglec F$^+$; and neutrophils, CD45$^+$CD11b$^+$MHC class II$^-$CD24$^+$Siglec F$^-$. Average ± standard deviation is shown (n=8-14 mice per group). * indicates a significant difference between groups, $P < 0.05$, by Student's *t*-test.

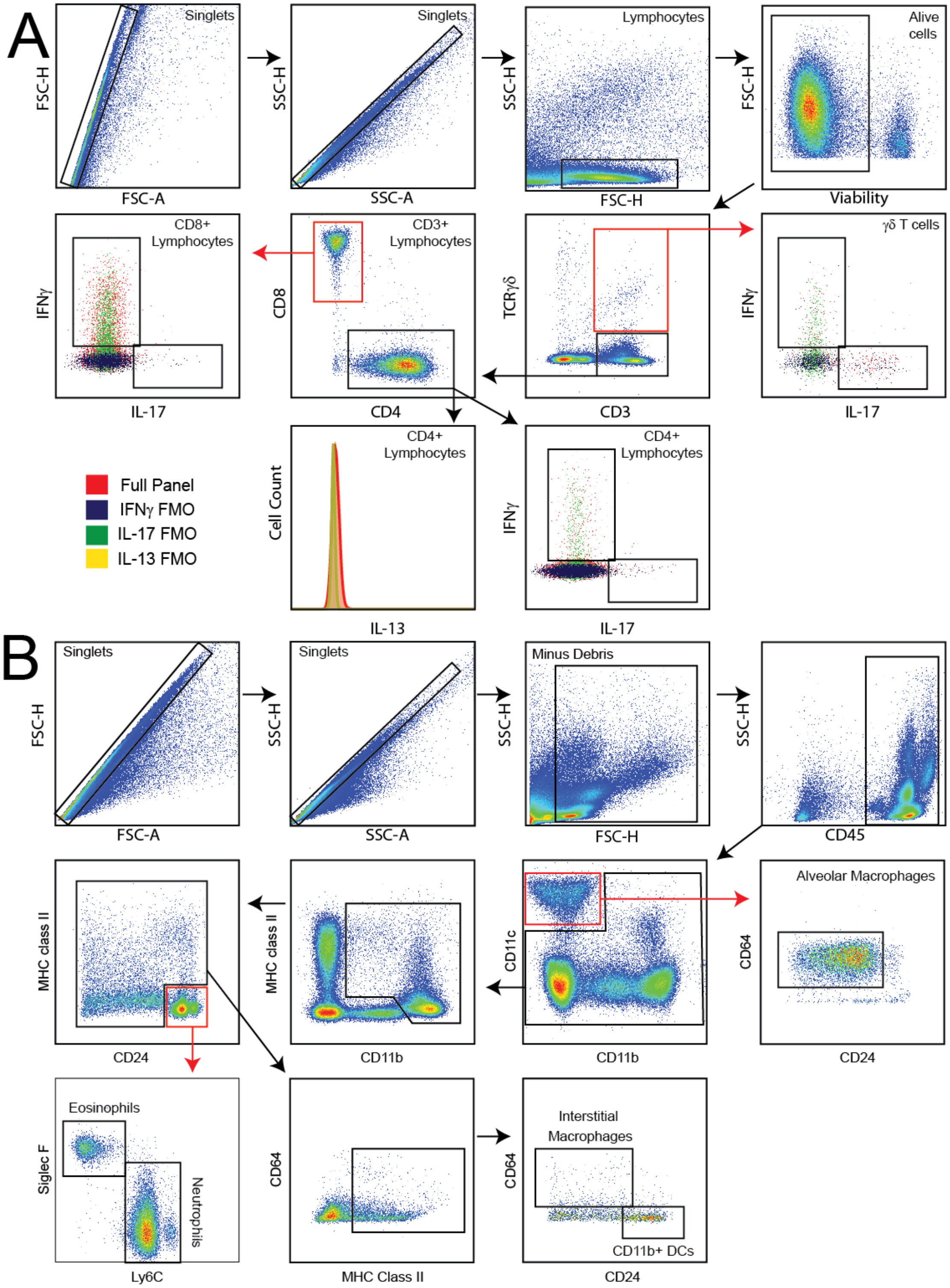


**S6. Figure. Gating strategies for lymphocyte and leukocyte profiling.** A) Sample gating strategy depicted with a wild-type untreated mouse with fluorescent minus one (FMOs) controls shown for IL-17, IFNγ and IL-13. B) Gating strategy for leukocyte profiling depicted with a wild-type untreated mouse. Macrophages and dendritic cells (DCs) were also selected for CD11c+ staining (not shown).
